# Perceived parental support in childhood and adolescence and suicidal ideation in young adults: a cross-sectional analysis of the i-Share study

**DOI:** 10.1186/s12888-018-1957-7

**Published:** 2018-11-27

**Authors:** Melissa Macalli, Marie Tournier, Cédric Galéra, Ilaria Montagni, Aicha Soumare, Sylvana M. Côté, Christophe Tzourio

**Affiliations:** 1 Inserm, Bordeaux Population Health Research Center, University of Bordeaux, UMR 1219, F-33000 Bordeaux, France; 2 Charles Perrens Hospital, Bordeaux, France; 3 University of Montreal, Montreal, Québec Canada; 4 University of Bordeaux, 146 rue Léo Saignat, 33076 Bordeaux Cedex, Bordeaux France

**Keywords:** Perceived parental support, Suicidal ideation, College students, Young adults, i-Share cohort

## Abstract

**Background:**

Suicidal ideation and suicidal risk assessment are major concerns for health professionals. The perception of a low level of parental support is a risk factor for suicidal tendencies among adolescents, but little is known about its long-term impact on the vulnerability to suicidal behavior in young adults. We investigated whether the perceived level of parental support during childhood and adolescence was associated with current suicidal ideation in young adults.

**Methods:**

We retrieved data collected in the i-Share study from February 1st, 2013 through January 30, 2017. This cross-sectional study included 10,015 French students, aged 18–24 years that completed an on-line self-reported questionnaire about suicidal ideation in the last 12 months and their perceived parental support in childhood and adolescence. We performed multinomial logistic regressions and sensitivity analyses to assess associations between the degree of perceived parental support and the frequency suicidal thoughts, after adjusting for the main known risk factors of suicidal ideation. We employed multiple imputations to account for missing data.

**Results:**

The study sample included 7539 female (75.7%) and 2436 male (24.3%) students (mean [SD] age 20.0 [1.8] years). About one in five students reported occasional suicidal thoughts (*n* = 1775, 17.7%) and 368 students (3.7%) reported frequent suicidal thoughts. The adjusted multinomial logistic regression revealed a significant negative association between perceived parental support and suicidal thoughts. A lack of perceived parental support in childhood and adolescence was associated with > 4-fold elevated risk of occasional (adjusted OR, 4.55; 95% CI: 2.97–6.99) and nearly 9-fold elevated risk of frequent (adjusted OR, 8.58; 95% CI: 4.62–15.96) suicidal thoughts, compared to individuals that perceived extremely strong parental support. This association was strongest among students with no personal history of depression or suicide attempts.

**Conclusions:**

Students that perceived low levels of past parental support had a higher risk of suicidal ideation. Past perceived parental support appeared to be a potent marker of suicidal risk in young adults. This marker should be routinely collected in studies on suicidal risk in young adults, and it could be considered an additional screening tool.

## Background

Suicide is the second leading cause of death among young adults between the ages of 15 and 29 years [1, 2], including college students [3]. The estimated prevalence of suicide ideation ranges from 6 to 12% among college students [4–7]. A recent meta-analysis pooled 36 college student cohorts (i.e., 634,662 students) and estimated that the 12-month prevalence of suicidal ideation was 10.6% (95% CI: 9.1–12.3). There was no significant difference between the prevalence estimates according to European and North American nationalities [8].

Suicidal ideation is common in young adults. It is the first step on the pathway to suicide [5] and one of the main risk factors for suicide attempts and suicides [9–11]. Suicidal behaviors are the result of complex interactions between social, psychological, and environmental risk factors. Moreover, many investigators have shown that, within this etiological heterogeneity, familial contributions are potent factors. Adoption and twin studies suggested that genetic factors account only in part for transmission of suicidal behavior [12]. Suicidal behaviors have also been associated with exposure of children and adolescents to domestic violence or sexual abuse, family conflicts, parent loss, parental divorce or separation, and a family history of mood disorder or substance abuse [5, 13–15].

Some studies have suggested that low levels of perceived parental support (PPS) were associated with higher suicidal ideation in adolescents [16–18], but little is known about this relationship in young adults. In this study, we tested the hypothesis that a low level of PPS in childhood and adolescence induced a persistent impact on the vulnerability to suicidal ideation in young adults.

## Methods

### Study population and data collection

Our study sample comprised participants of the ongoing, internet-based, Students’ Health Research Enterprise (i-Share) project, a prospective, population-based study of students in French-speaking universities and higher education institutions. Enrollment in the i-Share project started in 2013; to be eligible, a student had to be officially registered at a University or higher education institute, at least 18 years of age, able to read and understand French, and provide informed consent for participation. No compensation was given to students for completing the questionnaire on which the analyses of the current paper are based.

The i-Share protocol was approved by the *“Commission nationale de l’informatique et des libertés”* (CNIL- National Commission of Informatics and Liberties), which ensures that data collection does not violate freedom, rights, or human privacy. No information on ethnic or racial origin was collected in i-Share.

Students were informed about the purpose and aims of the study through flyers, communications in classes, social media, and a newsletter (http://www.i-Share.fr). After formal pre-registration on the i-Share online portal, a change of password, and validation of the informed consent, students completed the self-administered baseline questionnaire. This questionnaire recorded information on the participant’s health status, personal and family medical history, sociodemographic characteristics, and lifestyle habits.

For this cross-sectional study, we acquired data from a large sample of students that had participated in the i-Share cohort study between February 2013 and January 2017. Students were eligible only when they completed all items in the questionnaire. We restricted our analyses to students aged 18–24 years old, because this age range is defined as young adults, by the World Health Organization [19], and it is typically associated with major changes in a student’s life.

### Measures

#### Outcome: Suicidal thoughts

Suicidal thoughts were investigated with the question: *In the last 12 months, how often have you thought of committing suicide (had suicidal ideation)?* Participants selected one of three possible responses: (1) no suicidal thoughts, (2) occasional suicidal thoughts, and (3) frequent suicidal thoughts.

#### Exposure variable: Perceived parental support in childhood and adolescence

PPS was investigated with the question: *During your childhood and adolescence, how would you describe the support and comfort provided by your family?* Participants selected one of five different responses: (1) none, (2) low, (3) moderate, (4) strong, and (5) extremely strong.

#### Covariates

The following potentially confounding self-reported variables were considered in the analyses: age, gender (male, female), parents divorced or separated (yes, no), parental death (yes, no), did not live in parental home during childhood (yes, no), perceived economic status in childhood (adequate to very comfortable, difficult to very difficult), parental history of depression or anxiety (yes, no), and personal history of depression or attempted suicide (yes, no). Given the low number of students with a history of attempted suicide, the history of depression and attempted suicide were grouped into one variable.

### Statistical analyses

#### Descriptive analyses

We first described the variables in the whole sample and divided individuals into three groups, according to the frequency of suicidal thoughts. Continuous variables are expressed as the mean ± SD. Categorical variables are described as the proportion (range). The Kruskal-Wallis test was used to compare the distributions of age in the three groups of suicidal thoughts. Proportions were compared with the Chi-square test.

#### Model construction

Unadjusted and adjusted multinomial logistic regression models were utilized to assess the relationship between PPS and the frequency of suicidal thoughts. Model convergence was systematically checked. The assumption of linearity of the logit was tested for the continuous variable, age. We also tested interactions between PPS and the covariates included in the model. The fully adjusted complete-case analysis took into account age, gender, parental divorce or separation, parental death, not living in the parental home during childhood, perceived economic status in childhood, and a personal history of depression or attempted suicide. We also conducted a sensitivity analysis in another adjusted model, which included the additional factor: parental history of depression or anxiety.

#### Non-response and multiple imputation

Students were allowed to declare that they were not willing to answer certain questions considered sensitive, such as suicidal thoughts, PPS, personal history of depression or attempted suicide, parental divorce, parental death, and not living in the parental home in childhood. These refusals were coded as missing data. The literature shows the value of imputing in case of missing data rather than excluding them [20]. It also highlights the pitfalls associated with misuse of this method [21] such as making a false assumption of missingness at random. It is therefore advisable to present the two results of these analyses and to compare them. Thus, in the main analysis, participants with missing data were excluded in order to present a complete data analysis. Then we performed a sensitivity analysis with multiple imputation on missing data in order to take into account non-response among eligible students. We chose the method known as multiple imputation by chained equation (MICE). This method was based on an algorithm of the Monte Carlo Markov chain method, which was adapted for imputation on arbitrary or non-monotonic data [22]. First, we analyzed the shape of the refusal data; then we formulated the assumption of missing-at-random data. We hypothesized that the typology of the non-response data was informative, and that it did not occur completely at random, because it reflected the student’s unwillingness to answer. Therefore, according to this method, we assumed that all the information on the missing data was contained in the observed data. This method assumes that the distributions of the incomplete variables are conditional on the other variables of the imputation model. We performed 10 imputations. For each set of imputed data, we estimated the imputed model parameters by taking into account all variables of the chosen model.

#### Attributable fraction

The attributable fraction (AF) was defined as the proportion of suicidal thoughts attributable to PPS, calculated as follows [23]:

$$ \mathrm{AF}=\frac{p\left( RR\hbox{-} 1\right)}{p\left( RR\hbox{-} 1\right)+1} $$

where *p* represents the underlying prevalence of the risk factor (low PPS) in our population; i.e., the prevalence of low PPS levels (including questionnaire responses of moderate, low, or none) in our sample. *RR* is the relative risk of suicidal thoughts in the exposed population (i.e., the population that described PPS as moderate, low, or none) divided by the risk of suicidal thoughts in the unexposed population (the population that described PPS as extremely strong, or strong). This calculation was also used to determine the AF for other family events measured in the study.

All *p*-values were two-tailed, and *p* < 0.05 was considered statistically significant. Analyses were performed with SAS version 9.4 (SAS Institute Inc., Cray, NC, USA).

## Results

### Sample description based on suicidal thoughts

**Fig. 1:**
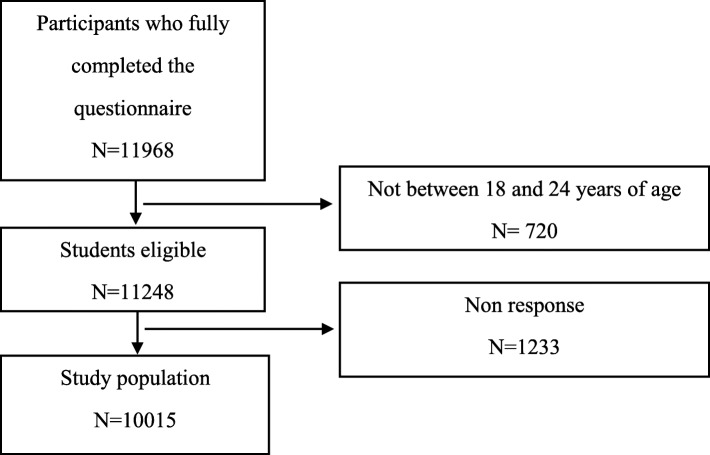
Flow chart of the study population (i-Share cohort, 2013–2017)

Of the 11,968 students that fully completed the questionnaire, 720 were excluded because they were not between 18 and 24 years of age. Another 1223 (11%) were excluded because they were not willing to answer questions related to suicidal thoughts, PPS, and confounding variables (Fig. 1). For all variables, excluded non-respondents comprised less than 4% (range: 1 to 4%). Participants were allowed to refuse to answer the question regarding parental history of depression or anxiety. In this case, a rather large number of individuals (*n* = 1045, 10%) refused to respond; therefore, we decided to include this group of participants in the analyses. The final study population included 10,015 students.

Compared to the 10,015 included participants, the 1233 excluded students that did not answer the questions of interest reported more occasional (*n* = 208, 25.6% vs. *n* = 1775, 17.7%) and frequent (*n* = 77, 9.5% vs. *n* = 368, 3.7%, *p* < 0.0001) suicidal thoughts. Additionally, compared to included students, the excluded students were more likely to describe PPS as “none” and to respond “yes” to a personal history of depression, attempted suicide, or negative childhood events (Table 1).

**Table 1:** Comparison of key variables between participants and nonparticipants*

Characteristic	Participants (*n* = 10,015)	Nonparticipants (*n* = 1233)	*p* ^**^
Age, mean (SD)	20.0 (1.8)	19.7 (1.7)	<.0001
Female, No. (%)	7579 (75.7)	935 (75.8)	0.904
Parental divorce or separation, No. (%)	3114 (31.1)	349 (40.4)	<.0001
Parental death, No. (%)	376 (3.8)	145 (14.2)	<.0001
Did not live in parental home during childhood, No. (%)	196 (2.0)	73 (6.1)	<.0001
Perceived economic status in childhood, No. (%)			<.0001
Adequate to very comfortable	9193 (91.8)	1024 (83.1)	
Difficult to very difficult	822 (8.2)	209 (17.0)	
Parental history of depression, No. (%)	3925 (43.8)	478 (54.2)	
Personal history of depression or attempted suicide, No. (%)	1274 (13.7)	242 (24.7)	<.0001
Suicidal thoughts, No. (%)			<.0001
No	7872 (78.6)	529 (65.0)	
Occasional	1775 (17.7)	208 (25.6)	
Frequent	368 (3.7)	77 (9.5)	
Perceived parental support, No. (%)			<.0001
Extremely strong	3277 (32.7)	250 (23.9)	
Strong	4065 (40.6)	372 (35.6)	
Moderate	1907 (19.0)	265 (25.3)	
Low	610 (6.1)	120 (11.5)	
None	156 (1.6)	39 (3.7)	

*Data are from the i-Share cohort, 2013–2017

^**^P-Values are based on Kruskall-Wallis test for continuous variables and the Chi^2^ test for categorical variables

In our sample, 7872 (78.7%) students reported no suicidal thoughts. About one student in five reported suicidal thoughts in the past 12 months; of these, 1775 (17.7%) had occasional suicidal thoughts and 368 (3.7%) had frequent suicidal thoughts.

Table 2 shows the sample characteristics, both overall and categorized by the type of suicidal thoughts. The mean age of the participants was 20.0 years (±1.8) and about three quarters were female (*n* = 7539; 75.7%). Participants with occasional and frequent suicidal thoughts were more likely to declare negative family events, such as parental divorce (*p* < 0.0001) or parental death (*p* = 0.0004), compared to those with no suicidal thoughts. They were more likely scholarship holder (*p* < 0.0001) and to declare an economic status in childhood as difficult to very difficult (*p* < 0.0001). Students that declared suicidal thoughts also reported more parental history of depression or anxiety than those with no suicidal thoughts. A personal history of depression or attempted suicide was reported by more than half the students with frequent suicidal thoughts (*n* = 216; 58.7%), about a third of those with occasional suicidal thoughts (*n* = 496; 27.9%), and less than 10% of those without suicidal thoughts (*n* = 662; 8.4%, *p* < 0.0001).

**Table 2:** Characteristics of the study sample categorized by the frequency of suicidal thoughts over the preceding year^*^

Characteristic	All students (*n* = 10,015)	No suicidal thoughts (*n* = 7872)	Occasional suicidal thoughts (*n* = 1775)	Frequent suicidal thoughts (*n* = 368)	*p* ^***^
Age, mean (SD)	20.0 (1.8)	20.0 (1.8)	20.0 (1.8)	19.9 (1.8)	0.5081
Female, No. (%)	7579 (75.7)	5926 (75.3)	1366 (77.0)	287 (78.0)	0.1895
Parental divorce or separation, No. (%)	3114 (31.1)	2342 (29.8)	633 (35.7)	139 (37.8)	< 0.0001
Parental death, No. (%)	376 (3.8)	274 (3.5)	75 (4.2)	27 (7.3)	0.0004
Did not live in parental home during childhood, No. (%)	196 (2.0)	136 (1.7)	45 (2.5)	15 (4.1)	0.0010
Perceived economic status in childhood, No. (%)					< 0.0001
Adequate to very comfortable	9193 (91.8)	7297 (92.7)	1730 (97.5)	353 (95.9)	
Difficult to very difficult	822 (8.2)	575 (7.3)	191 (10.8)	56 (15.2)	
Scholarship, No. (%)	3810 (38.0)	2904 (36.9)	745 (42.0)	161 (43.8)	< 0.0001
Half time or more job activities, No. (%)	590 (5.90)	447 (5.7)	123 (6.9)	20 (5.4)	< 0.0001
Parental history of depression or anxiety, No. (%) ^**^	3925 (43.8)	2835 (39.8)	885 (57.7)	205 (65.3)	< 0.0001
Personal history of depression or attempted suicide, No. (%)	1374 (13.7)	662 (8.4)	496 (27.9)	216 (58.7)	0.1204
Perceived parental support, No. (%)					< 0.0001
Extremely strong	3277 (32.7)	2835 (36.0)	382 (21.5)	60 (16.3)	
Strong	4065 (40.6)	3275 (41.6)	693 (39.0)	97 (26.4)	
Moderate	1907 (19.0)	1336 (17.0)	450 (25.4)	121 (32.9)	
Low	610 (6.1)	354 (4.5)	194 (11.0)	62 (16.9)	
None	156 (1.6)	72 (0.9)	56 (3.2)	28 (7.6)	

*Data are from the i-Share cohort, 2013–2017

^**^ *n* = 8970, due to missing data

^***^P Values are based on Kruskall-Wallis test for continuous variable and Chi^2^ test for categorical variables

The proportion of participants that reported strong PPS (including extremely strong and strong) declined as the frequency of suicidal thoughts increased (*p* < 0.0001). Conversely, the proportion of participants that reported weak PPS (including questionnaire responses of moderate, low, and none) increased with increases in the frequency of suicidal thoughts. Thus, a total lack of PPS was more common among participants with frequent suicidal thoughts (*n* = 28; 7.6%) than among those with occasional suicidal thoughts (*n* = 56, 3.2%) or those without suicidal thoughts (*n* = 72, 0.9%).

### Association between PPS in childhood and adolescence and suicidal thoughts over the preceding year

Table 3 summarizes the unadjusted and adjusted multinomial logistic model results. We observed a negative association between PPS and suicidal thoughts. The unadjusted univariate multinomial logistic regression also showed that lower levels of PPS were associated with higher frequencies of suicidal thoughts. Moreover, having a total lack of PPS (questionnaire response: ‘none’) strongly increased the odds of having suicidal thoughts occasionally (OR, 5.77; 95% CI: 4.00–8.32) and frequently (OR, 8.38; 95% CI: 11.08–30.48), compared to having an extremely strong PPS (Table 3).

**Table 3:** Associations between perceived parental support in childhood and adolescence and the frequency of suicidal thoughts over the preceding year^*^

Perceived parental support	Unadjusted (*n* = 10,015)		Adjusted ^**^ (*n* = 10,015)		Adjusted ^***^ (*n* = 8970)	
	Occasional suicidal thoughts	Frequent suicidal thoughts	Occasional suicidal thoughts	Frequent suicidal thoughts	Occasional suicidal thoughts	Frequent suicidal thoughts
	OR (95% CI)	OR (95% CI)	OR (95% CI)	OR (95% CI)	OR (95% CI)	OR (95% CI)
Extremely strong	1.00	1.00	1.00	1.00	1.00	1.00
Strong	1.57 (1.37–1.80)	1.40 (1.01–1.94)	1.55 (1.35–1.78)	1.35 (0.96–1.88)	1.55 (1.34–1.79)	1.23 (0.86–1.74)
Moderate	2.50 (2.15–2.91)	4.27 (3.12–5.87)	2.26 (1.93–2.64)	3.40 (2.44–4.74)	2.11 (1.78–2.49)	2.90 (2.05–4.11)
Low	4.08 (3.31–5.00)	8.28 (5.70–12.00)	3.39 (2.73–4.19)	5.35 (3.60–7.95)	3.33 (2.64–4.20)	4.91 (3.23–7.47)
None	5.77 (4.00–8.32)	18.38 (11.08–30.48)	4.43 (3.02–6.49)	9.81 (5.60–17.19)	4.55 (2.97–6.99)	8.58 (4.62–15.96)

*OR* odds ratio, *CI* confidence interval.

*Data from the i-Share cohort, 2013–2017.

**Adjusted for age, gender, parental divorce or separation, parental death, not living in parental homeduring childhood, perceived economic status in childhood, and personal history of depression or attemptedsuicide.

***Adjusted for age, gender, parental divorce or separation, parental death, not living in parental home during childhood, perceived economic status in childhood, personal history of depression or attemptedsuicide, and parental history of depression or anxiety

A multiple adjustment to the model reduced the estimated values, but they remained relatively high. Thus, a total lack of PPS showed aORs of 4.43 for occasional suicidal thoughts (95% CI: 3.02–6.49) and 9.81 for frequent suicidal thoughts (95% CI: 5.60–17.19). In all models, we observed that the PPS was negatively associated with the risk of suicidal thoughts. Further adjusting with the parental history of depression or anxiety did not change the general interpretation of the results (Table 3).

We tested interactions between gender and perceived parental support and it was not significant (*p* for interaction = 0.40). To investigate whether there was a statistically significant interaction between PPS and a personal history of depression or attempted suicide, we performed a stratified regression analysis. The risks of both occasional and frequent suicidal thoughts increased with decreases in the level of PPS in both strata, but the risks were higher among students without a personal history of depression or attempted suicide (*p* for interaction < 0.001; Table 4). For example, a total lack of PPS was associated with a 6-fold risk of frequent suicidal thoughts (aOR, 6.01; 95% CI: 2.65–14.00) among students with a personal history of depression or attempted suicide, but the risk was eight-fold (aOR, 8.06; 95% CI: 2.92–22.23) for students without a personal history of depression or attempted suicide.

**Table 4:** Associations between perceived parental support in childhood and adolescence and suicidal thoughts over the preceding year, categorized by whether individuals had a personal history of depression or attempted suicide^*^

Perceived parental support	Personal history of attempted suicide or depression				No personal history of attempted suicide or depression			
	Adjusted ^**^ (*n* = 1374)		Adjusted ^***^ (*n* = 1193)		Adjusted ^**^ (*n* = 8641)		Adjusted ^***^(*n* = 7777)	
	Occasional suicidal thoughts	Frequent suicidal thoughts	Occasional suicidal thoughts	Frequent suicidal thoughts	Occasional suicidal thoughts	Frequent suicidal thoughts	Occasional suicidal thoughts	Frequent suicidal thoughts
	OR (95% CI)	OR (95% CI)	OR (95% CI)	OR (95% CI)	OR (95% CI)	OR (95% CI)	OR (95% CI)	OR (95% CI)
Extremely strong	1.00	1.00	1.00	1.00	1.00	1.00	1.00	1.00
Strong	1.18 (0.86–1.61)	1.64 (1.00–2.69)	1.22 (0.88–1.70)	1.54 (0.92–2.60)	1.68 (1.44–1.97)	1.01 (0.63–1.63)	1.67 (1.41–1.97)	0.91 (0.56–1.48)
Moderate	1.27 (0.90–1.77)	2.68 (1.62–4.38)	1.31 (0.92–1.88)	2.34 (1.38–3.99)	2.63 (2.20–3.15)	3.54 (2.26–5.57)	2.38 (1.97–2.89)	3.06 (1.92–4.86)
Low	1.75 (1.14–2.67)	4.00 (2.28–7.15)	2.00 (1.26–3.17)	4.26 (2.31–7.85)	4.19 (3.28–5.36)	5.88 (3.35–10.34)	3.97 (3.04–5.18)	4.93 (2.70–9.01)
None	1.62 (0.82–3.22)	6.46 (3.03–13.77)	1.85 (0.88–3.90)	6.01 (2.65–14.00)	6.33 (4.05–9.88)	10.29 (4.28–24.76)	6.45 (3.90–10.66)	8.06 (2.92–22.23)

*OR* odds ratio, *CI* confidence interval

^*^Data from the i-Share cohort, 2013–2017

^**^Adjusted for age, gender, parental divorce or separation, parental death, not living in parental home during childhood, perceived economic status in childhood, and perceived parental support × personal history of depression or attempted suicide

^***^Adjusted for age, gender, parental divorce or separation, parental death, not living in parental home during childhood, perceived economic status in childhood, perceived parental support × personal history of depression or attempted suicide, and parental history of depression or anxiety

When the model was tested after adding multiple imputations of non-response data, we found that the relative efficiency of the imputation on each of the variables was greater than 95%. This finding indicated that the number of imputations was sufficient for the fraction of non-response data. The estimations obtained from the imputed model were close to those obtained from the non-imputed model (Table 5). The association between PPS and suicidal thoughts was statistically significant for all models (*p* < 0.05) except in the multiple imputation models for the students without history of depression or suicide attempt.

**Table 5:** Associations between perceived parental support and suicidal thoughts in an adjusted multinomial logistic regression model after multiple imputation for non-response data^*^

Perceived parental support	Adjusted ^**^ (*n* = 11,248)		Adjusted model ^***^(*n* = 11,248)			
			Personal history of depression or attempted suicide		No personal history of depression or attempted suicide	
	Occasional suicidal thoughts	Frequent suicidal thoughts	Occasional suicidal thoughts	Frequent suicidal thoughts	Occasional suicidal thoughts	Frequent suicidal thoughts
	OR (95% CI)	OR (95% CI)	OR (95% CI)	OR (95% CI)	OR (95% CI)	OR (95% CI)
Extremely strong	1.00	1.00	1.00	1.00	1.00	1.00
Strong	1.49 (1.30–1.70)	1.30 (0.96–1.76)	1.17 (0.72–1.90)	1.53 (0.52–4.51)	1.60 (1.38–1.86)	1.10 (0.72–1.68)
Moderate	2.13 (1.83–2.47)	2.92 (2.15–3.96)	1.32 (0.78–2.24)	2.51 (0.86–7.34)	2.40 (2.02–2.86)	3.05 (2.00–4.66)
Low	3.05 (2.49–3.73)	5.17 (3.60–7.41)	1.64 (0.81–3.35)	4.07 (1.15–14.43)	3.74 (2.91–4.80)	5.86 (3.48–9.85)
None	4.07 (2.85–5.80)	8.54 (5.10–14.31)	1.69 (0.53–5.40)	5.93 (0.87–9.97)	5.68 (3.75–8.61)	8.83 (3.90–20.03)

*OR* odds ratio, *CI* confidence interval

^*^Data from the i-Share cohort, 2013–2017

^**^Adjusted for age, gender, parental divorce or separation, parental death, not living in parental home during childhood, perceived economic status in childhood, personal history of depression or attempted suicide, and parental history of depression or anxiety

***Adjusted for age, gender, parental divorce or separation, parental death, not living in parental home during childhood, perceived economic status in childhood, perceived parental support × personal history of depression or attempted suicide, and parental history of depression or anxiety

We also found that the risks associated with PPS were higher than the risks associated other negative childhood events measured in our study (Table 6), independent of the adjustment. This was confirmed by the calculation of attributable risk. We found that 20.5% of occasional and frequent suicidal thoughts could be attributed to an insufficient PPS (frequency of PPS rated ‘moderate’, ‘low’, or ‘none’ in our sample: 26%), which was the same percentage (20.5%) attributed to a personal history of depression or attempted suicide (frequency of personal history of depression or attempted suicide in our sample: 13.7%). Both these percentages were notably higher than the percentage of suicidal thoughts attributed to parental divorce (6.9%) or parental death (1.1%).

**Table 6:** Association between student characteristics and suicidal thoughts^*^

Characteristic	Unadjusted (*n* = 10,015)		Adjusted ^**^ (*n* = 8970)	
	Occasional suicidal thoughts	Frequent suicidal thoughts	Occasional suicidal thoughts	Frequent suicidal thoughts
	OR (95% CI)	OR (95% CI)	OR (95% CI)	OR (95% CI)
Age	1.01 (0.98–1.04)	0.97 (0.92–1.03)	0.99 (0.96–1.02)	0.91 (0.85–0.97)
Gender				
Male	1.00	1.00	1.00	1.00
Female	1.10 (0.97–1.24)	1.16 (0.90–1.50)	1.01 (0.88–1.16)	0.82 (0.62–1.10)
Parental divorce or separation				
No	1.00	1.00	1.00	1.00
Yes	1.31 (1.17–1.46)	1.43 (1.16–1.78)	0.89 (0.78–1.01)	0.81 (0.62–1.05)
Parental death				
No	1.00	1.00	1.00	1.00
Yes	1.22 (0.94–1.59)	2.20 (1.46–3.31)	1.08 (0.80–1.45)	1.75 (1.07–2.88)
Did not live in parental home during childhood				
No	1.00	1.00	1.00	1.00
Yes	1.48 (1.05–2.08)	2.42 (1.40–4.17)	1.07 (0.72–1.58)	1.21 (0.63–2.32)
Perceived economic status in childhood				
Correct to very comfortable	1.00	1.00	1.00	1.00
Difficult to very difficult	1.53 (1.29–1.82)	2.28 (1.70–3.07)	1.10 (0.90–1.35)	1.21 (0.63–2.32)
Parental history of depression or anxiety				
No	1.00	1.00	1.00	1.00
Yes	2.06 (1.84–2.31)	2.84 (2.24–3.60)	1.59 (1.41–1.80)	1.59 (1.22–2.08)
Personal history of depression or attempted suicide				
No	1.00	1.00	1.00	1.00
Yes	4.22 (3.71–4.81)	15.48 (12.40–19.33)	3.63 (3.13–4.20)	11.68 (9.06–15.07)
Perceived parental support				
Extremely strong	1.00	1.00	1.00	1.00
Strong	1.57 (1.37–1.80)	1.40 (1.01–1.94)	1.55 (1.34–1.79)	1.23 (0.86–1.74)
Moderate	2.50 (2.15–2.91)	4.27 (3.12–5.87)	2.11 (1.78–2.49)	2.90 (2.05–4.11)
Low	4.08 (3.31–5.00)	8.28 (5.70–12.00)	3.33 (2.64–4.20)	4.91 (3.23–7.47)
None	5.77 (4.00–8.32)	18.38 (11.08–30.48)	4.55 (2.97–6.99)	8.58 (4.62–15.96)

*OR* odds ratio, *CI* confidence interval

^*^Data from the i-Share cohort, 2013–2017

^**^Adjusted for age, gender, parental divorce or separation, parental death, not living in parental home during childhood, perceived economic status in childhood, personal history of depression or attempted suicide, and parental history of depression or anxiety

## Discussion

In this cross-sectional study on a large sample of 10,015 students, we found a strong association between PPS in childhood and adolescence and suicidal thoughts in young adults. Lower levels of PPS were associated with a higher frequency of both occasional and frequent suicidal thoughts. Thus, a total lack of PPS was associated with more than 4-fold increased risk of occasional suicidal thoughts (aOR, 4.55; 95% CI: 2.97–6.99) and nearly 9-fold increased risk of frequent suicidal thoughts (aOR, 8.58; 95% CI: 4.62–15.96). In all models, we observed a negative association between the level of PPS and the frequency of suicidal thoughts. Sensitivity analyses modifying adjustment and multiple imputation modeling provided consistent results.

Few studies have described the association between PPS and suicidal thoughts in young adults, and most did not control for confounding factors related to the family environment. In one study that included 5183 Chinese students, suicidal ideation was associated with poor family structures and relationships or improper parenting styles [24]. In another study that included 188 African American students, strong family support was associated with a lower incidence of suicide ideation [25]. Similarly, in a Taiwanese study that included 2919 college students, a positive linear trend was observed between increased suicidal tendency and a parenting style with low affection [26]. In a younger age range (adolescents 12 to 18 years old), it was shown that inadequate social and family support increased the risk of suicide or suicidal ideation [27–31]. Similarly, a cross-sectional study that included 448 adolescents aged 13 to 17 years measured PPS with the Perceived Social Support from Family Scale. In that study, each one point increase in PPS was associated with a 54% lower frequency in suicidal plans [32]. Compared to the present study, all those previous studies were more focused on current parental support, in addition to other social determinants. However, a longitudinal study conducted among a large sample of adolescents showed conflicting results. Parental support was predictive of lower levels of depression but was not significantly associated with the outcomes related to suicidal behaviors [33].

The present study had some important strengths, including the size of the sample, the strength of the associations, the PPS-dose-dependent pattern, the consistency of our results with previous studies, and the large number of variables collected and adjusted for in the multivariable models. Furthermore, when we performed multiple imputation for non-response data and the sensitivity analysis, we found consistent results. However, there were also some limitations in this study. First, it was a cross-sectional analysis; therefore, we could not strictly separate the timing of exposure, outcome, and covariates. Moreover, no causality could be inferred between PPS and suicidal ideation. Second, only brief and succinct measures could be done in large sample studies and perceived parental support as well as suicidal ideation were assessed with only one item. This is a limitation that has to be taken into account when interpreting our results. However, the prevalence of suicidal thoughts found in our study fall within the range reported by the main studies on the subject [4–6, 8] which was somehow reassuring. Third, the voluntary participation of students may have introduced a self-selection bias, although it is difficult to see how this potential bias could have influenced the observed associations. Fourth, the information was self-reported, which could lead to an information bias, particularly if participants under-reported the frequency of suicidal thoughts or the presence of a personal and/or family history, due to considerations of social acceptability. Again, this under-reporting would be expected to have a low impact on the associations observed. Fifth, there is an over-representation of women in our sample compared to the 56% of female students in France. However, we tested interactions with gender for the main analyses and none was significant. Further, in stratified analyses, aOR did not differ significantly between males and females and the confidence intervals were largely overlapping (data not shown). Finally, although we had information on confounding factors, we could not rule out the influence of residual or unmeasured confounding factors, due to the complexity of the suicidal thought process. In addition, because our predictor variable was PPS, a recall bias might have led to an overestimation of the associations between support and suicidal ideation. However, our findings on the differential roles that PPS played for students with and without a history of depression suggested that a recall bias might have had limited effect. Indeed, we postulated that, if recall bias was a major source of influence, the association between low support and suicidal thoughts would be stronger in students with a history of depression compared to those without. Instead, we found the reverse; students without a history of depression were more likely to report suicidal thoughts, when they also reported low parental support during childhood and adolescence.

We found that our estimation of the risk associated with PPS was higher than previous estimated risks of other variables known to be associated with suicidal ideation, such as parental divorce [34, 35] or parental death [36, 37]. We also noted that the specific role of low PPS could not be distinguished from the roles of other negative parenting practices, such as abuse or neglect (not measured in our study). However, our associations remained significant after controlling for other negative childhood events, such as parental death or divorce.

The association between PPS and suicidal thoughts could reflect a familial aggregation of suicidal thoughts and mood disorders. Contributors to his type of aggregation might be unknown psycho-social, clinical, or biological factors, including genetic factors [38]; for example, parents that provide low support might be experiencing depression [39].

Our results in young adults, if confirmed by other studies, could eventually lead to the development of intervention programs for families at an early stage of life and thus be a prerequisite for more targeted, less costly and more effective prevention interventions [40]. In adolescents, such programs have yielded promising results to decrease the incidence of suicidal thoughts in young adults after an intervention started in adolescence [41, 42]. These interventions, aimed primarily at building parenting support and supervision capacities, are strategies developed by the CDC in suicide prevention [43]. Other programs such as attachment-based family therapy [44] aim to transform the quality of adolescent-parent attachment in order to provide the adolescent with a safer relationship that can support him during difficult times and crises related to suicidal thoughts and behaviors.

Regardless of the subjectivity of the PPS variable or the etiology of suicidal thoughts, evaluating PPS could be useful in assessments of suicidal risk in young adults. Given that PPS is a relatively neutral, non-intrusive variable, health professionals can readily assess PPS to improve suicide risk screening. Our findings indicated that PPS could be a particularly important marker, because the association between PPS and suicidal ideation was stronger in the absence than in the presence of a personal history of attempted suicide or depression. This is remarkable, because a personal history of attempted suicide or depression is an important marker of suicidal risk. Our findings highlighted the importance of interventions that aim to screen for and correct risky situations that children might face at home.

To summarize, our results indicated that a low PPS in childhood and adolescence was strongly associated with frequent suicidal thoughts in young adults. This issue should be systematically addressed in further clinical studies on suicidal risk in young people and, if confirmed, it could be considered in routine care. Longitudinal studies should assess the ability of PPS to predict the risk of suicide attempts and suicide.

## Abbreviations

AF: Attributable fraction
CI: Confidence interval
CNIL: Commission nationale de l’informatique et des libertés - National Commission of Informatics and Liberties
i-Share: Internet-based Students’ Health Research Enterprise
MICE: Multiple imputation by chained equation
OR: Odds ratio
PPS: Perceived parental support
RR: Relative risk
SD: Standard deviation
WHO: World health organization
